# Harnessing machine learning to explore influencing mechanism in the dual pro-environmental intention-behavior gap

**DOI:** 10.1038/s41598-026-42468-1

**Published:** 2026-03-05

**Authors:** Zihao Dong, Yu Zhang, Yanying Mao, Liudan Jiao, Xiaosen Huo, Liu Wu

**Affiliations:** 1 School of Economics and Management, Chongqing Jiaotong University, Chongqing, 400074 China; 2 Chongqing Key Laboratory of Computational Intelligence, Key Laboratory of Big Data Intelligent Computing, College of Computer Science and Technology, Chongqing University of Posts and Telecommunications, Chongqing, 400065 China; 3 Department of Communication Engineering, Chongqing College of Electronic Engineering, Chongqing, 401331 China

**Keywords:** Machine learning, Pro-environmental behaviors, Intention-behavior gap, Color-code model, Environmental social sciences, Psychology, Psychology

## Abstract

Fostering pro-environmental behaviors (PEBs) is crucial for advancing low-carbon development. A significant obstacle in this endeavor is the intention-behavior gap, where intentions fail to translate into actual behaviors. This study addresses both the commonly discussed negative gap, where high intentions do not lead to corresponding behaviors, and the less explored positive gap, where behaviors exceed intentions. Drawing from 2216 questionnaires, this study compared eight machine learning methods and selected LightGBM as the optimal approach. And the study examined the impact of individual and situational factors on these two types of gaps by LightGBM. The results identify robust predictive associations: for Cooperative-Grey-PEBs, attitude and ascription of responsibility exhibit inverted U-shaped patterns, while high environmental knowledge supports behavior maintenance in no-intention contexts. For Negative-Grey-PEBs, the negative gap narrows when attitude surpasses a critical threshold (4.5). Furthermore, higher levels of ascription of responsibility and self-efficacy are associated with a lower negative gap. Conversely, high infrastructure visibility is characterized by a divergent pattern, where it correlates with an expanded negative gap, consistent with a “responsibility dilution effect”. The study proposes tailored measures for different groups, which would have significant implications for policies aiming to bolster low-carbon development.

**Supplementary Information:**

The online version contains supplementary material available at https://doi.org/10.1038/s41598-026-42468-1.

## Introduction

The increasing frequency and severity of extreme weather events have made the transition toward low-carbon development more urgent than ever^1,2^. Recent reports indicate that climate change has intensified heatwaves, droughts, and other extreme events across most global regions since the mid-20th century, resulting in substantial economic and social losses, particularly in climate-vulnerable economies^3^. The widespread and rapid increase in greenhouse gases is leading to intensified climate change, already impacting natural disasters^4^. Simultaneously, natural disasters are contributing to a rise in carbon emissions, creating a harmful cycle^5,6^. To address these issues and minimize socio-economic damage, it’s crucial to transition towards low-carbon solutions.

Advancing PEBs is a crucial step towards achieving the low-carbon transition. These behaviors encompass actions aimed at amplifying positive impacts or mitigating negative ones on the environment^7–9^. For instance, proper recycling practices contribute to resource conservation and the reduction of environmental contamination. Agovino et al.^10^, Shruti^11^, Tumu^12^, Yang^13^,. Similarly, the practice of sorting waste is acknowledged as an effective approach to tackling solid waste problems and advancing circular economic development, environmental protection, and public health^14^. Additionally, adopting energy-efficient appliances plays a vital role in facilitating the low-carbon transition. Such appliances consume less electricity^15^, which not only lowers household carbon emissions but also lessens the overall energy demand^16,17^. Furthermore, there would be a profound emission reduction impact when promoting PEBs across billions of individuals^18^.

Most prior research has concentrated on identifying the determinants of pro-environmental behaviors (PEBs), often grounded in the Theory of Planned Behavior (TPB)^19–21^. Based on TPB, scholars have affirmed the impact of attitudes^22^, subjective norms^23,24^, and perceived behavior control on intentions to engage in PEBs^25^. For instance, He et al.^26^, found that environmental communication is a useful tool to foster pro-environmental intentions. Shanmugavel and Balakrishnan^27^, highlighted the significant role of personal norm in shaping positive behavioral intention towards e-vehicles. Gansser and Reich^28^, highlighted that the attitude toward sustainable behavior significantly impacts intention.

Three research gaps exist in prior studies that utilized the TPB to examine PEBs and their intentions. The first gap lies in the TPB’s omission of other potential factors influencing PEBs. Several studies verified that individual and external factors had impacts on PEBs beyond the scope of TPB. The analysis of factors influencing the intention-behavior gap in PEBs often overlooks the simultaneous consideration of individual and external factors. Initial studies concerning individual-level influences highlighted demographic variables, including gender, age, educational attainment, marital status, living location, and economic background^29,30^. For example, Thøgersen and Grønhøj^31^, found that women tended to practice energy-saving behaviors more often than men in daily life. In terms of external factors, Steg and Vlek^30^, indicated that pro-environmental intentions and actions are substantially shaped by contextual factors such as the presence of recycling infrastructure, the quality of public transit, and the availability of low-carbon goods.

The second gap is that most previous studies have neglected to consider the disparity between intention and behavior when examining PEBs using TPB. The overlooked aspect of the intention-behavior gap in pro-environmental behaviors is its dural nature. Current research on the intention-behavior gap primarily addresses the phenomenon where high pro-environmental intentions fail to translate into corresponding behaviors. Conversely, the phenomenon of low intentions associated with high behaviors has received minimal attention. Instances where actions occur without prior intentions can create a distinct type of intention-behavior gap, characterized by high levels of PEBs and low levels of intentions^32^. This form of intention-behavior gap is termed as “positive intention-behavior gap” in this study. Conversely, when there are high levels of intentions but low levels of PEBs, it constitutes the “negative intention-behavior gap”. This study adopted the color-coded behavioral model to gain a deeper understanding of the intention-behavior gap, categorizing individuals into four distinct types based on levels of intention and behavior^33^.

Thirdly, prior studies on pro-environmental behaviors and intention–behavior gap have relied primarily on regression-based models and structural equation modeling grounded in the Theory of Planned Behavior. While these approaches are theoretically robust, they typically impose linear or weakly nonlinear assumptions and are less suited to capturing complex interaction structures and heterogeneous patterns across multiple behavioral configurations. This study used machine learning as a new analytical tool to deal with these problems. Importantly, the use of machine learning in this study is not intended to establish causal relationships. Instead, it adopts a predictive and exploratory perspective to identify systematic association patterns that can inform theory development and generate hypotheses for future experimental or longitudinal research.

The significance of the present paper is threefold. First, it offers an innovative extension of the Theory of Planned Behavior (TPB) to gain a more holistic understanding of the factors that influence individuals’ PEBs. Second, the paper introduces a dual categorization of the intention-behavior gap, presenting it as either a positive or negative gap. This approach allows for a comprehensive investigation of the dynamics between intentions and behaviors. Third, this study applied multiple machine learning methods to the pro-environmental intention-behavior gap to cope with the limitations of traditional statistical methods in identifying non-linear relationships, thus predicting the formation mechanism of pro-environmental intention-behavior separation more precisely.

The paper is organized as follows: Sect. 2 introduces the theoretical foundation and constructs a color-coded model of pro-environmental behaviors (PEBs). Section 3 explains the research methods. Section 4 presents the findings, which are then discussed in Sect. 5. Finally, Sect. 6 concludes the study and highlights relevant policy recommendations.

## Theoretical model

### The extended theory of planned behavior

Ajzen^34^, proposed the Theory of Planned Behavior (TPB), which suggests that attitudes, subjective norms, and perceived behavioral control influence intentions, subsequently guiding behavior. Over the last three decades, TPB has become a foundational model for understanding pro-environmental behaviors^35^. It is widely applied to analyze determinants of environmental actions, including low-carbon travel^36^, recycling practices^37^, water-saving efforts^38^, energy reduction behaviors^39^, and sustainable consumption^40^.

Nevertheless, TPB does not fully account for situational and personal factors that may affect intentions, behaviors, and the gap between them. To enhance its ability to predict pro-environmental actions, researchers have extended the model by incorporating variables like past behavior^41^, moral norms^42^, and self-identity^43^. These extensions have improved the model’s effectiveness in forecasting pro-environmental intentions and behaviors.

The framework is based on the Theory of Planned Behavior, with additional variables included to capture relevant psychological dimensions, rather than to formally integrate multiple theories.

### Individual factors

The first individual factor influencing PEBs is knowledge, which refers to a person’s awareness and understanding of environmental issues. According to the knowledge deficit model^44^, individuals are more likely to perform PEBs when they have sufficient knowledge about environmental problems^45,46^, especially concerning the scientific causes and consequences of these issues^47,48^. Another important factor is the ascription of responsibility, defined as the degree to which an individual feels personally accountable for the environmental harm caused by their actions^49^. A stronger sense of personal responsibility generally encourages greater engagement in PEBs^50–52^. The third factor involves emotions, encompassing the positive or negative feelings individuals associate with PEBs. Engaging in pro-environmental activities can elicit positive emotions such as pride^53^ or a warm glow^54,55^, which enhance self-satisfaction. Because individuals naturally seek positive emotional experiences, the anticipation of such self-focused positive feelings is associated with greater engagement in PEBs^53,56,57^.

Although these factors are important for pro-environmental behaviors, they have rarely been examined together. Hence, this study intends to analyze them simultaneously to better understand the causes of the intention-behavior gap and help narrow it.

### Situational factor

Situational factor primarily includes infrastructure, which refers to the systems and facilities that impact people’s PEBs, such as transportation systems, public service facilities, and similar structures. Azhgaliyeva et al.^58^, posited that enhancing infrastructure has a positive influence on the selection of PEBs. The availability of convenient transportation, well-established recycling facilities, or other infrastructure supporting specific PEBs in a region may positively or negatively affect individuals’ willingness to adopt corresponding behaviors. This can further extend considerations of behavior controllability and implementation. Including the situational factor enriches our approach to PEBs and strengthens the theory’s practical relevance and explanatory capacity. This relatively comprehensive consideration is crucial for accurately explaining and predicting individual PEBs. It contributes to a deeper comprehension of decision-making across diverse scenarios and clarifies the underlying causes of the intention-behavior gap.

**Fig. 1:**
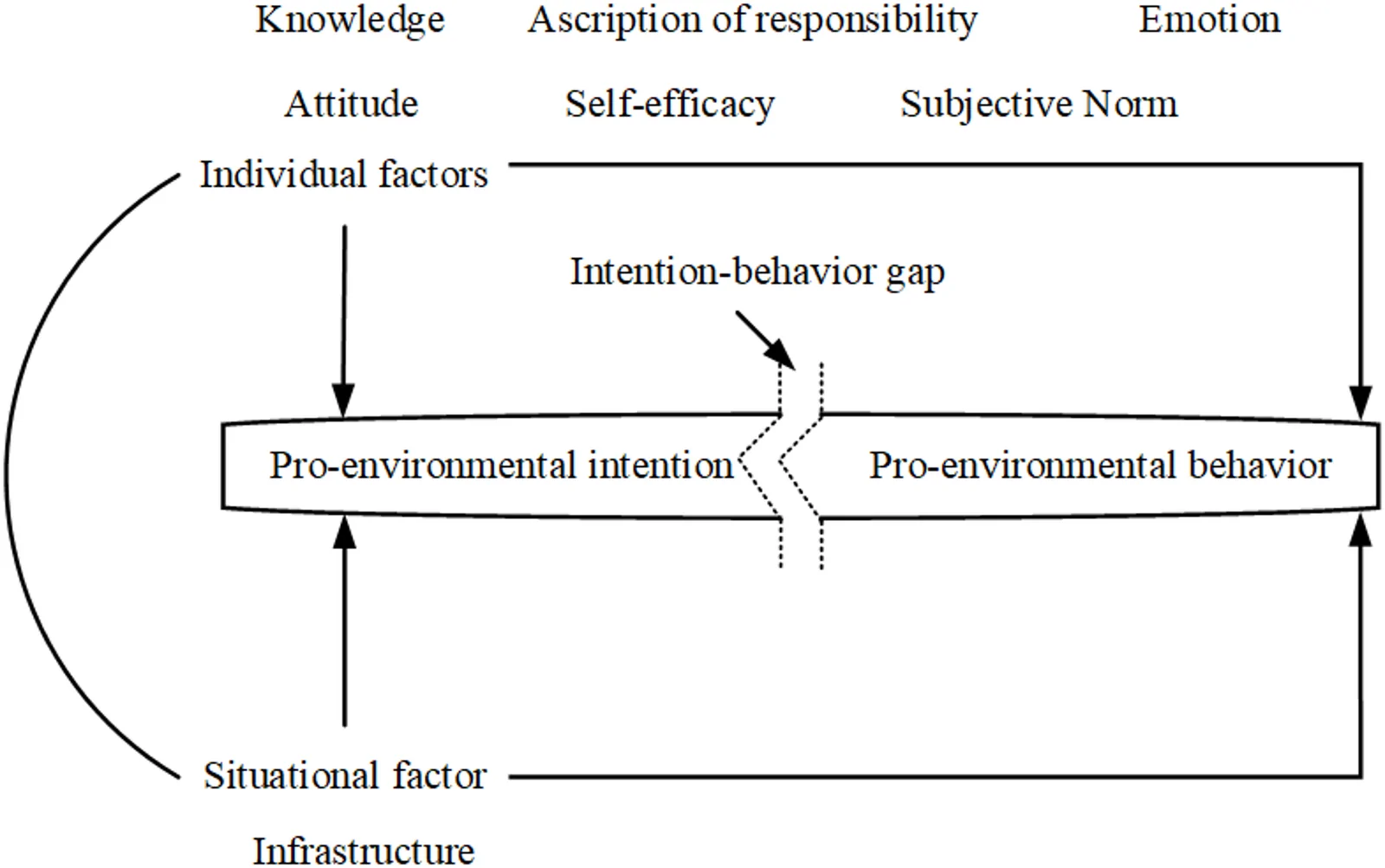
Conceptual framework of the research.

Thus, we extended TPB by adding situational factors and individual factors, drawing inspiration from the conceptual framework proposed by Barr et al.^59^. for extending the theory of rational behavior. This extension results in a more comprehensive conceptual framework (see Fig. 1). Through this framework, our goal is to explore how these factors influence PEBs, intentions, and the intention-behavior gap. The specific definitions for each factor can be found in Table 1.

**Table 1:** The definitions of the factors influencing PEBs.

variables	Theoretical framework	Definition
Knowledge	Knowledge deficit model	Understanding environmental issues involves recognizing their causes, impacts, related policies, and potential solutions
Ascription of responsibility	Norm activation model	Degree of responsibility of individuals for the environmental consequences of their actions
Emotion	Norm activation model	Emotional responses of people to their environmental behavior
Subjective Norm	Theory of planned behavior	The perception individuals have regarding the expectations and pressures placed upon them within their social environment.
Self-efficacy	Theory of planned behavior, Protection motivation theory	The perception of one’s own competence and effectiveness in performing actions required to attain desired outcomes.
Attitude	Theory of planned behavior	An individual’s favorable or unfavorable assessment of a particular behavior.
Infrastructure		Infrastructure refers to the systems and facilities that impact people’s pro-environmental behaviors, such as transportation systems, public service facilities, and similar structures.

## Methodology

### Variables measurement

#### Dependent variable

 PEBs are commonly defined as deliberate actions intended to minimize negative environmental impacts^60,61^. Researchers have further classified PEBs into two primary types: habit-adjustment behaviors and interpersonal facilitation behaviors^62^. Accordingly, this study focuses on these two dimensions for measurement. Habit-adjustment behaviors include routine, environmentally friendly practices—for example, turning off lights, computers, or televisions after use. In contrast, interpersonal facilitation behaviors reflect efforts to influence others, such as actively promoting environmental awareness and encouraging eco-friendly actions within households, workplaces, and communities. Both behavioral categories were assessed using Likert-scale items (1 = strongly disagree, 5 = strongly agree). To evaluate pro-environmental intentions (PEIs), two statements were used: “I am willing to…” and “I intend to…”. Full details of the items used to measure PEBs and PEIs are provided in Table S1.1 (Supplementary Material).

**Fig. 2:**
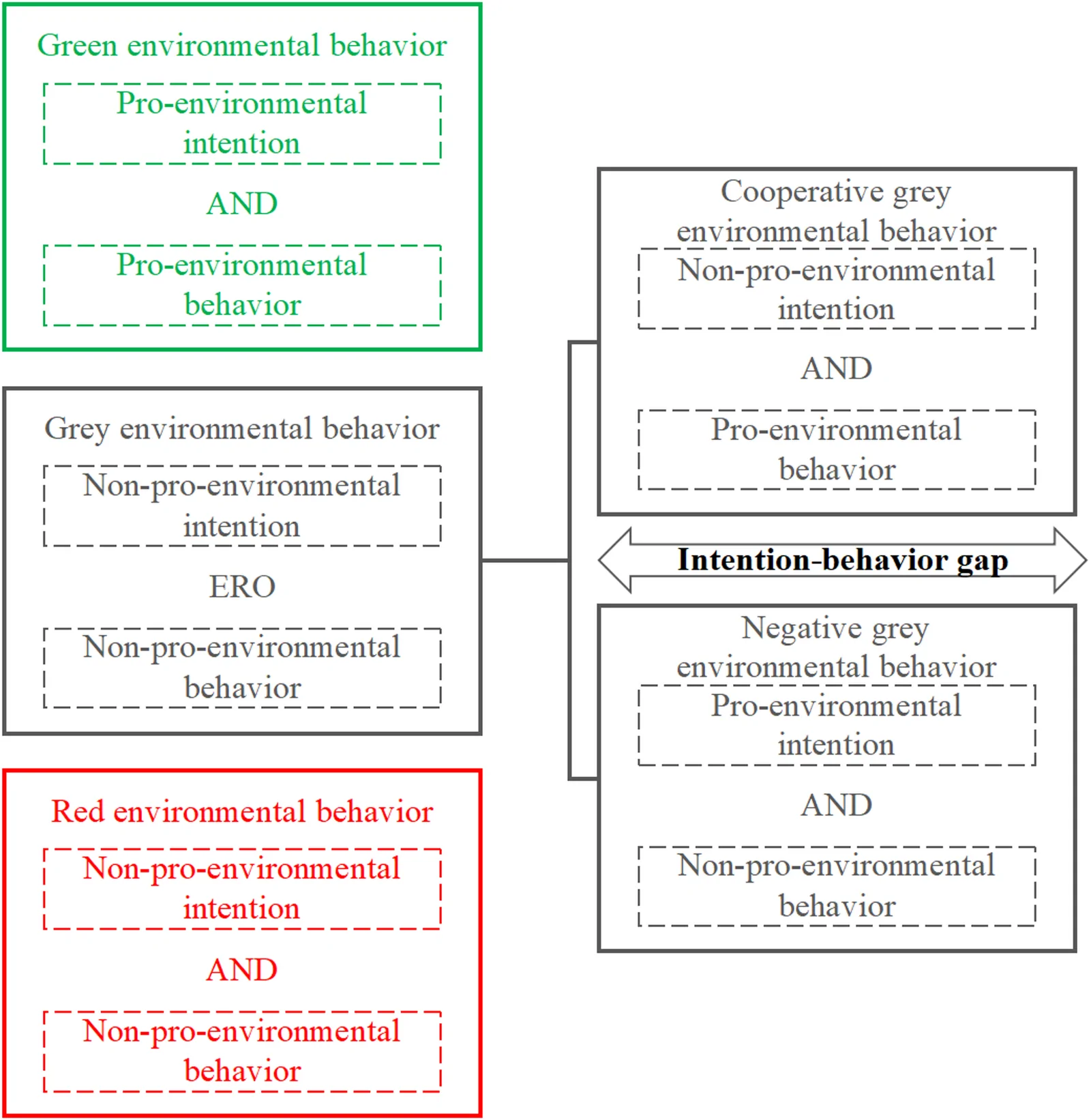
Three colors of pro-environmental behaviors.

This study adapted the framework introduced by Geng et al.^33^, and applied it to the context of PEBs. Based on this model, PEBs were classified into three categories—green, red, and gray—in order to systematically explore the various influencing factors behind each type. The objective of this approach is to capture the complexity and heterogeneity of PEBs and to offer a deeper explanation of their underlying dynamics. By examining the causes of the gap between intention and behavior, the model aims to generate insights and practical suggestions for narrowing this divide. The meanings associated with each PEB category are illustrated in Fig. 2.

Below are the methods used to quantify various types of PEBs.

In our study, we employed the median as a methodological tool to classify behaviors and intentions, following the approach suggested by^63^. We acknowledge that the use of a median split may lead to information loss and classification noise; however, it is adopted here as a pragmatic operationalization consistent with the categorical structure of the color-coded behavior framework. Specifically, we analyzed 2216 data points to determine the median value for PEBs, which was found to be 4. We defined this median value, denoted as *F*, as the threshold for categorizing PEBs. Participants with *F* values greater than 4 were classified as exhibiting PEBs, while those with *F* values of 4 or less were categorized as exhibiting non-PEBs (Eq. (1).

1 $$Behaviormode=\left\{\begin{array}{c}PEBsifF>4\\Non-PEBsifF\le4\end{array}\right.$$

Following the same analytical approach for PEBs, we applied the median method to classify pro-environmental intentions (*F’*) among the 2216 data points. The median value for pro-environmental intentions was also determined to be 4. Respondents whose *F’* larger than 4 are classified as having pro-environmental intentions while whose *F’* less than or equal to 4 are classified as having non-pro-environmental intentions.

2 $$Pro-environmentalintentions=\left\{\begin{array}{c}Pro-environmentalintentionsifF{\prime}>4\\Non-pro-environmentalintentionsifF{\prime}\le4\end{array}\right.)$$

As per the theoretical model of color-coded PEBs depicted in Fig. 1, Green-PEBs, $\mathrm{Cooperative}$-Grey-PEBs, $\mathrm{Negative}$-Grey-PEBs, and Red-PEBs (the dependent variable) were respectively assigned values of 1, 2, 3, or 4 (Eq. (3)). The numerical values assigned to the four categories serve only as labels for classification and do not represent an ordinal scale.

Color coded pro-environmental behaviors

3 $$\left\{\begin{array}{l}\mathrm{\:Green\:pro-environmental\:behavior}\mathrm{s}\mathrm{\:(assigned\:1)\:if\:}F>4\mathrm{\:and\:}F{\prime}>4\\\mathrm{\:Cooperative\:grey\:pro-environmental}\text{}\mathrm{behavior}\mathrm{s}\mathrm{\:(assigned\:2)\:if\:}F>4\mathrm{\:and\:}F{\prime}\le4\\\mathrm{\:Negative\:grey\:pro-environmental\:behavior}\mathrm{s}\mathrm{\:(assigned\:3)\:if\:}F\le4\mathrm{\:and\:}F{\prime}>4\\\mathrm{\:Red\:pro-environmental\:behavior}\mathrm{s\:}\mathrm{(assigned}\mathrm{\:4)\:if\:}F\le4\mathrm{\:and\:}F{\prime}\le4\end{array}\right.$$

#### Independent factors

The independent variables in this study were primarily measured based on established scales drawn from prior research, including Liu et al.^64^,, Nurul Alam et al.^65^,, Ertz et al.^66^,, Zelenski and Desrochers^67^. A five-point Likert scale was employed, with responses ranging from 1 (strongly disagree) to 5 (strongly agree), reflecting the degree of agreement with each item. Detailed measurement items and their corresponding sources are provided in Table S1.1 of the Supplementary Material.

### Data collection

The finalized questionnaire was conducted between March and August 2023, following refinement based on a preliminary pilot survey. The survey was distributed online via the professional platform “Questionstar” and social media channels like WeChat. To ensure the sample represented diverse demographics across all provinces of China, a snowball sampling technique was adopted. Initially, the questionnaire link was shared with existing contacts and social networks, who were then encouraged to forward it to others likely to be interested. In total, 2,407 responses were collected. After filtering out responses completed in under 90 s or exhibiting careless answers, 2,216 valid questionnaires remained, resulting in an effective response rate of 92.1%.

### Machine learning model building and evaluation

To thoroughly capture the heterogeneous mechanisms underlying different PEB groups, this study utilizes eight machine learning algorithms: Decision Tree (DT), Random Forest (RF), XGBoost, LightGBM, Lasso, Multilayer Perceptron (MLP), K-Nearest Neighbors (KNN), and Multinomial Logistic Regression (MLR). Initially, Boruta was applied for feature selection. Subsequently, the dataset was randomly split into training and testing sets with a 70:30 ratio, using a fixed random seed of 4321 to guarantee result reproducibility. For details of the method, see S2 in Supplementary Material.

**Fig. 3:**
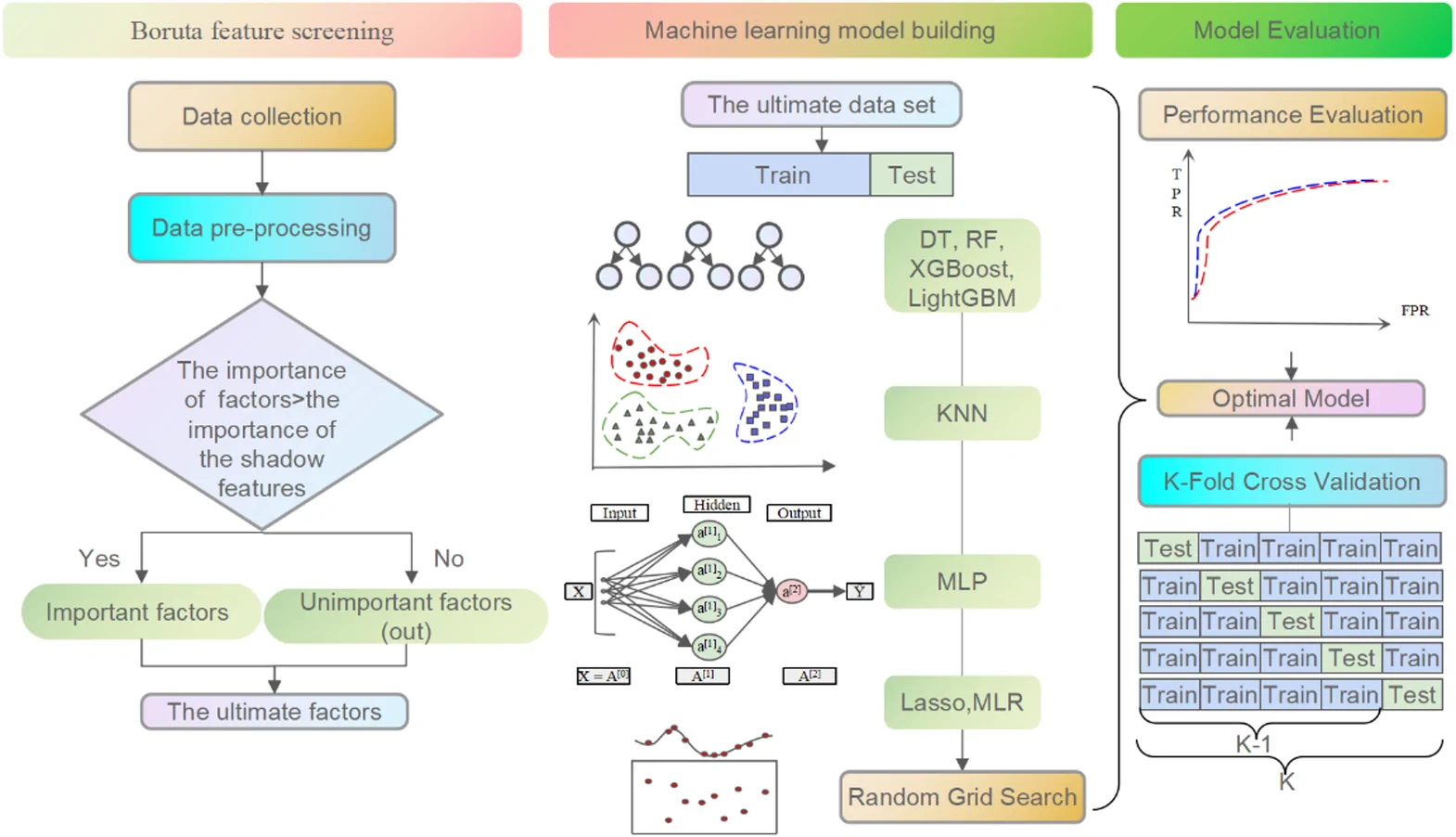
Research flowchart.

Prior to model evaluation, Hyperparameter tuning was conducted using random grid search within the training set and 5-fold cross-validation were conducted for each algorithm to ensure robust and reliable outcomes. The optimal model was selected based on ROC AUC scores and cross-validation performance. Data preprocessing, model development, and analysis were implemented using specialized software to enhance accuracy and efficiency. Specifically, data preprocessing was performed with R version 4.4.2, while model building employed the “tidymodels” package in R 4.2.2. Additionally, the “fastshap” package in R 4.4.2 was used to compute SHAP values, facilitating interpretation of feature contributions and visualization through SHAP plots. The detailed workflow is illustrated in Fig. 3.

## Results

### Reliability and validity test

Table 2 presents the results for average variance extracted (AVE) and composite reliability (CR). According to Malhotra and Dash^60^, AVE thresholds can be overly stringent, and reliability may be adequately established by CR alone. In this study, the minimum CR value was 0.667, surpassing the recommended cutoff of 0.6^68^, indicating strong internal consistency of the questionnaire items. Although some constructs exhibit composite reliability values close to the lower acceptable threshold, these measures are retained given the exploratory nature of the study and their theoretical relevance. Furthermore, all AVE values listed in Table 2 exceeded the suggested benchmark of 0.5^69^, demonstrating satisfactory construct validity of the measurement instrument. Formal discriminant validity tests were not the primary focus of this study, therefore, potential conceptual overlap among related constructs should be considered when interpreting the results.

**Table 2:** Scale reliability and validity test.

Variables	CR	AVE
Behavior	0.811	0.591
Intention	0.909	0.833
Knowledge	0.761	0.548
Ascription of responsibility	0.843	0.642
Self-efficacy	0.719	0.561
Attitude	0.882	0.788
Subjective Norm	0.747	0.599
Emotion	0.667	0.516
Infrastructure	0.813	0.604

_Note: CR, composite reliability; AVE, average variance extracted_.

### Multiple collinearity test

**Table 3:** Diagnosis of Multicollinearity Among Independent Variables.

Independent Variables	VIF
Knowledge	1.319
Ascription of responsibility	2.699
Self-efficacy	2.250
Attitude	2.618
Subjective norm	2.188
Emotion	1.952
Infrastructure	1.796

Table 3 presents the results of the multicollinearity diagnostics, the Variance Inflation Factor (VIF) for each independent variable is strictly below 5, indicating the absence of multicollinearity and confirming the independence of these variables. This ensures the validity of the regression analysis results.

### Demographics and clusters of color-coded behaviors

 Table S1.2 in the Supplementary Material presents the demographic characteristics of the participants. According to the table, there is an almost equal distribution of gender in the survey sample, with males accounting for 43.5% and females for 56.5%. In terms of age, a substantial majority (70.4%) of respondents are young and middle-aged adults under 35 years old. The educational background of the participants is varied and evenly spread out among different levels. These sociodemographic attributes suggest that the survey respondents represent a diverse group, which provides a solid basis for examining pro-environmental intentions and behaviors among urban residents in China.

**Fig. 4:**
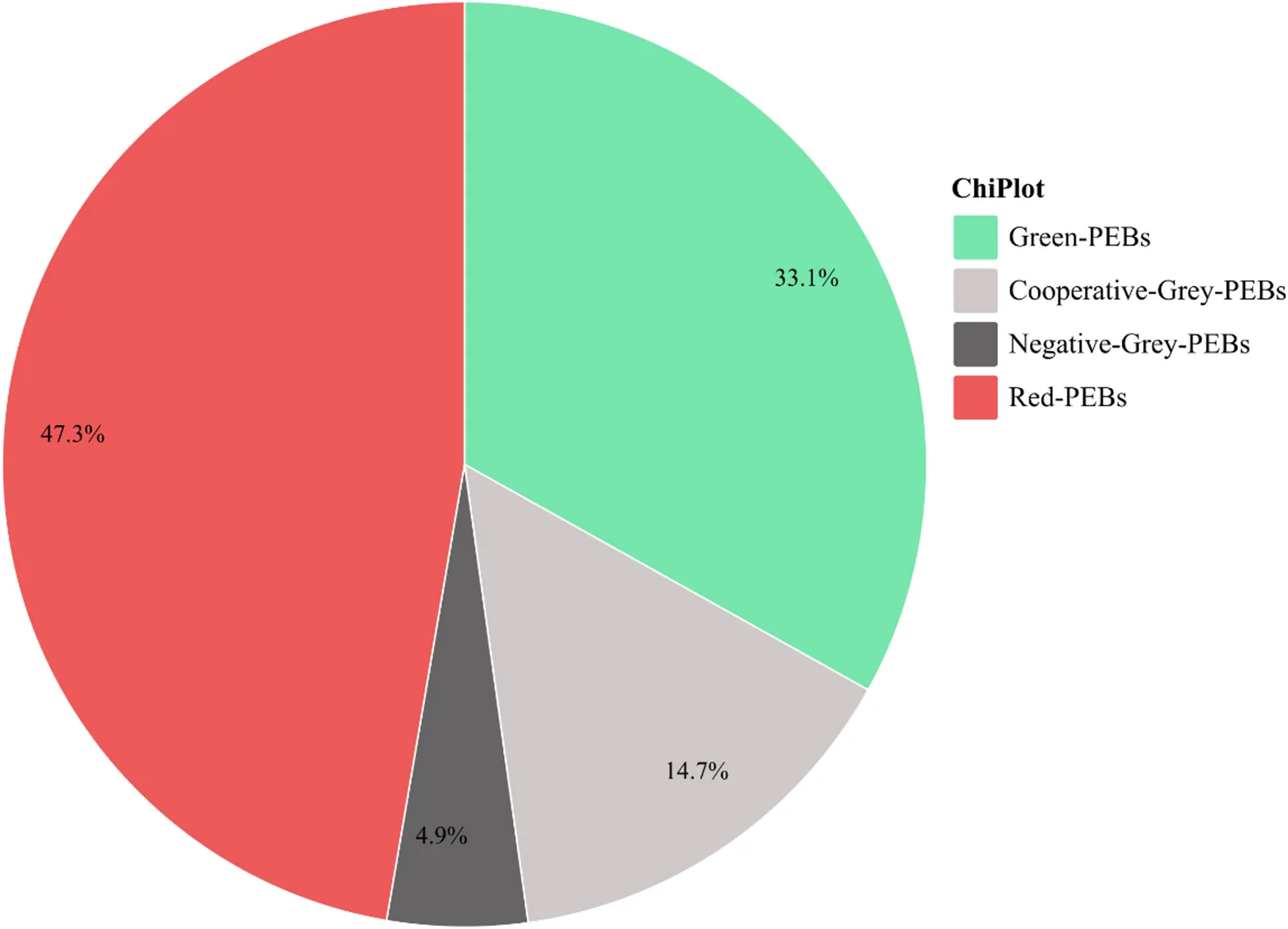
Percentages of four clusters of color-coded behavior among 2216 samples.

Figure 4 presents a pie chart depicting the distribution of the four clusters of color-coded PEBs among the 2216 samples. Figure 3 clearly depicts that based on our categorization, Red-PEBs represents the largest percentage, followed by Green-PEBs, Cooperative -Grey-PEBs, and Negative -Grey-PEBs respectively. It is interesting to observe that 19.6% of the respondents exhibit an intention-behavior gap, with 14.7% of individuals showing less drive for PEBs based on intentions and 4.9% failing to translate their intentions into PEBs. This finding highlights the need to not only focus on individuals’ intentions but also on the barriers and obstacles preventing them from translating those intentions into PEBs.

### Boruta feature selection

**Fig. 5:**
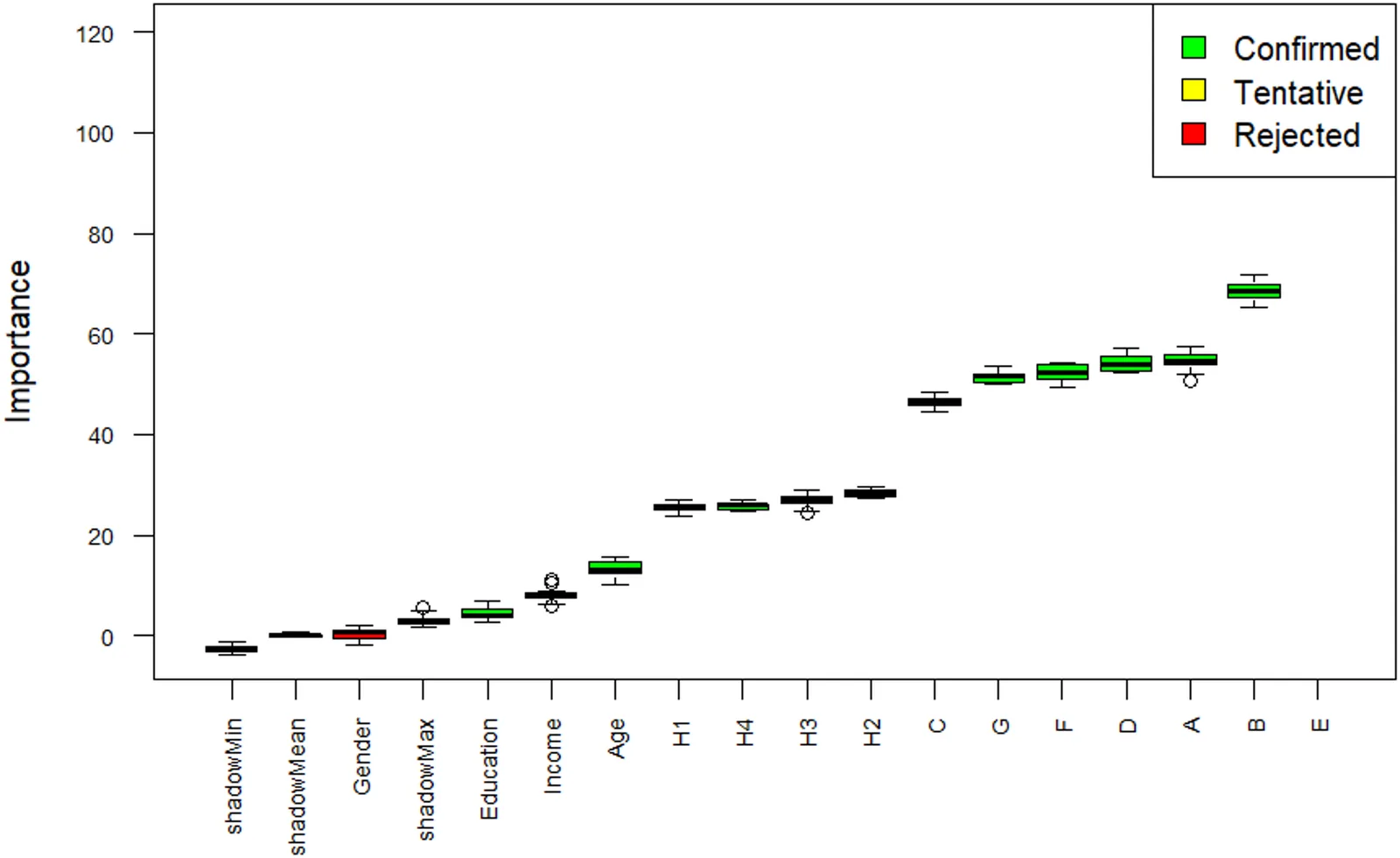
Boruta feature importance ranking and feature selection details.

The Boruta feature selection method is a fully-variable feature selection method designed to evaluate the importance of each feature and determine its contribution to a machine learning model^70^. Boruta evaluates the importance of each feature by comparing its importance to a set of randomly generated “shadow” features. Each feature is categorized as “important” (green), “unimportant” (red), and “uncertain” (yellow), with different colors or markers representing different categories.

The advantage of the Boruta method is that it can effectively identify all the features in the data that contribute significantly to the target variable without missing important features. Compared to traditional feature selection methods such as recursive feature elimination (RFE) or univariate feature selection, Boruta is more comprehensive.

 As shown in Fig. 5 and Table S1.3, only the gender variable did not pass the test, so gender needed to be removed from subsequent analyses. This indicates that through the Boruta screening method, we have successfully identified and retained the most representative features in the dataset. These features will help to improve the predictive performance of the model while reducing redundant features and avoiding model overfitting.

### Machine learning comparison

**Fig. 6:**
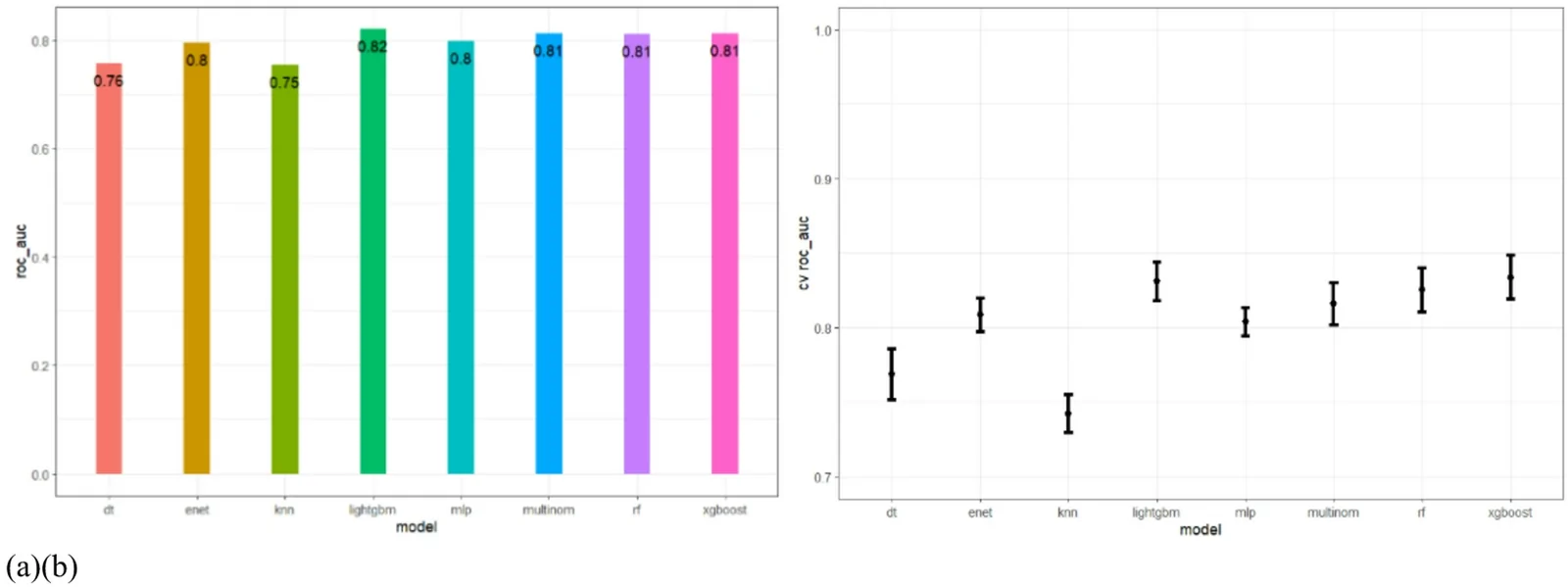
Column plots of ROC-AUC values for each model on the test set and averages of metrics for cross-validation of each model (with upper and lower limits).

The results of the comparison of the eight machine learning models are shown in Fig. 6. Since the proportional distribution of the dependent variables in this study is not balanced, we also performed smote and upsample oversampling, but the results obtained were generally inferior to the original data, with serious overfitting and underfitting problems, so we finally decided to use the original data for the analysis.

As can be seen from Fig. 6(a), the lightgbm model has the highest roc_auc value, indicating that it has the best performance, and as can be seen from Fig. 6(b), the lightgbm has the highest mean value of roc_auc and a narrow confidence interval, indicating that it has excellent generalization ability and stability. In summary we choose lightgbm for the following analysis.

### Machine learning analytics

**Fig. 7:**
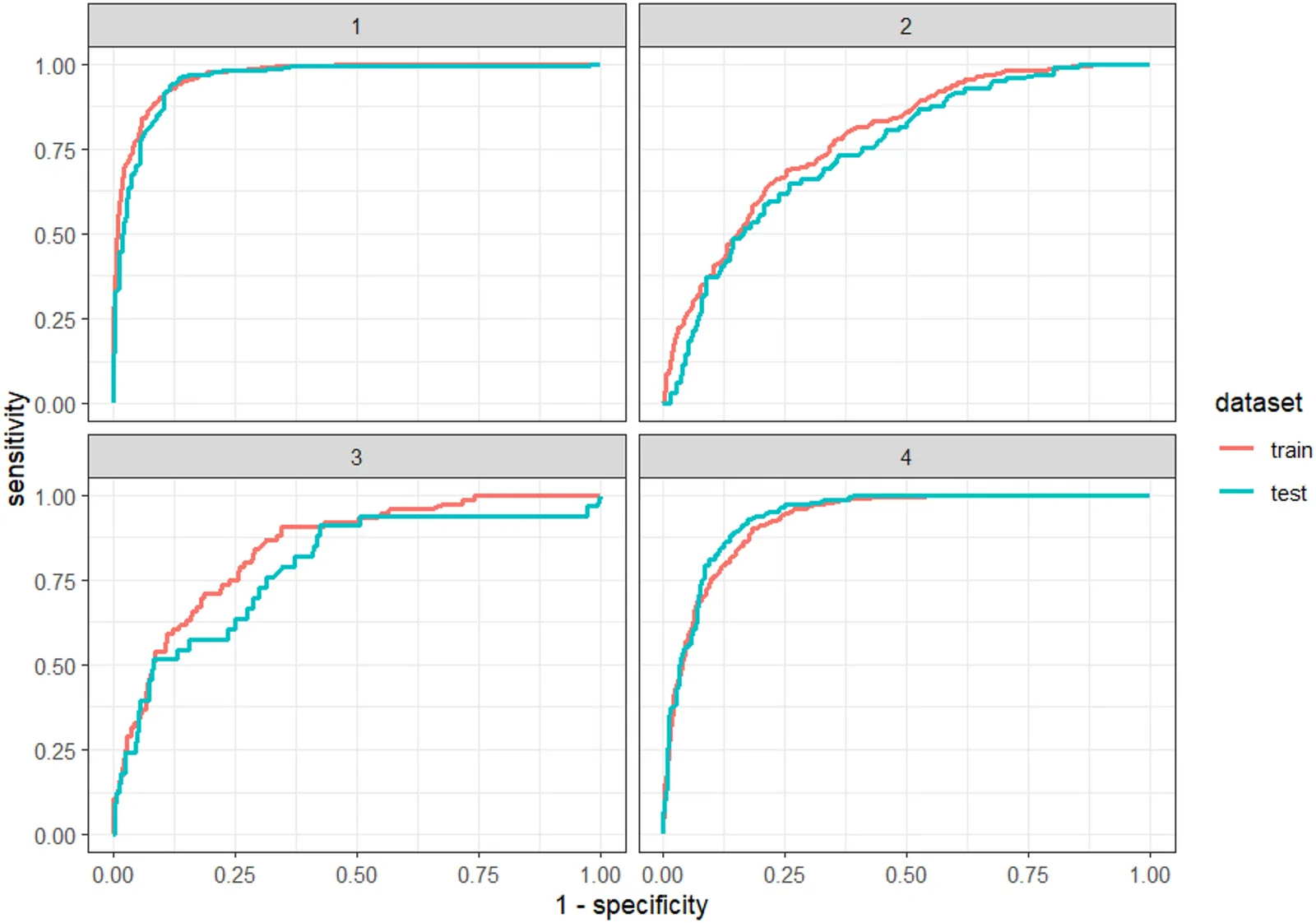
ROC-AUC curve for lightgbm.

**Table 4:** Performance evaluation metrics for lightgbm.

Train	AUC	Accuracy	F1	Precision	Recall
	0.85	0.76	0.57	0.52	0.47
Test	0.82	0.76	0.85	0.77	0.47

In this study, we used the lightgbm model to analyze the data and assessed its performance by AUC, Accuracy, F1, Precision and Recall (see Table 4) and the ROC-AUC curve is shown in Fig. 7.

The model predicts category 1 best, followed by categories 4, 3, and 2. The overall AUC values are 0.85 (training set) and 0.82 (test set), and the value of Accuracy is 0.76 on both training set test sets, which makes the model’s performance more stable. However, the values of F1 and Precision on the training set are smaller than those on the test set, the main reason is that there is a category imbalance on the training set, while the imbalance is reduced on the test set due to the small amount of data. Since this study focuses on the model differentiation ability rather than on the category imbalance problem, it mainly focuses on AUC and Accuracy, the values of which indicate that the model has good prediction and generalization ability. Lower recall values for certain categories indicate reduced predictive accuracy for these groups, which should be considered when interpreting class-specific results.

**Fig. 8:**
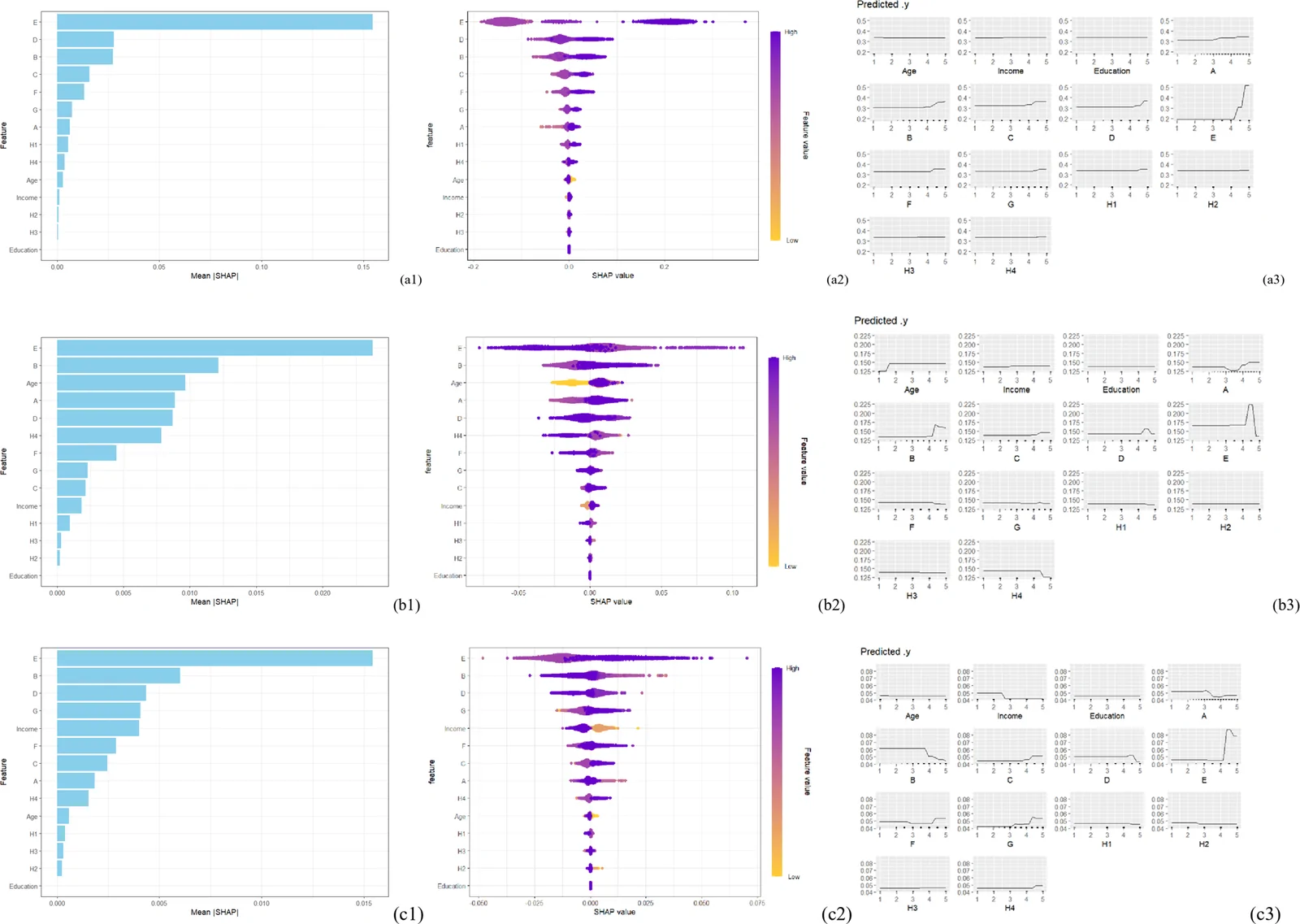

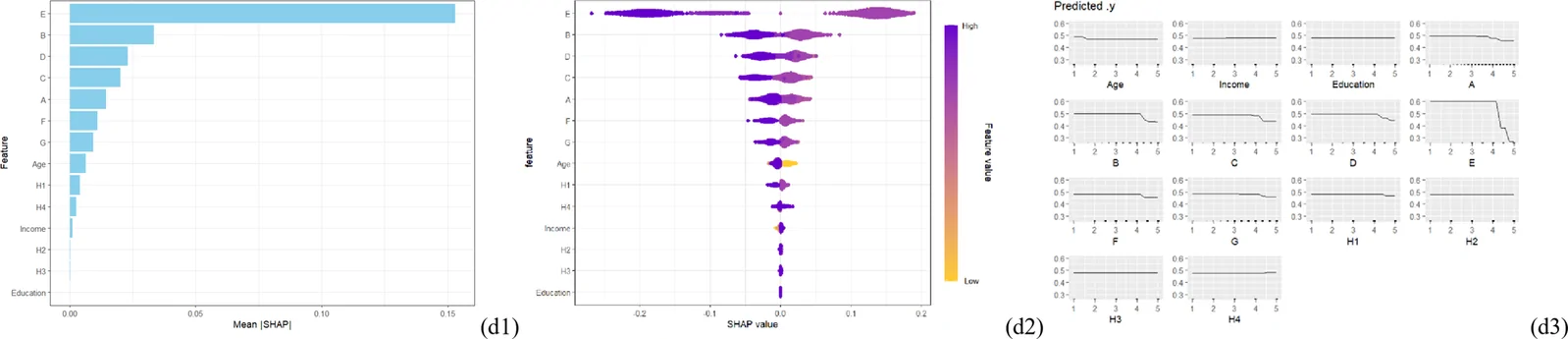
SHAP importance ranking, SHAP swarm and partial dependency graph Notes: (a) for Green-PEBs, (b) for Cooperative-Grey-PEBs, (c) for Negative-Grey-PEBs, (d) for Red-PEBs.

For Green-PEBs, as shown in Fig. 8(a1), the four variables with the highest importance are, in order, attitude (E), self-efficacy (D), ascription of responsibility (B), and subjective norm (C). Figures 8(a2) and 8(a3) show that higher values of attitude are associated with higher predicted probabilities of Green-PEBs when the value exceeds 4, while self-efficacy, ascription of responsibility, and subjective norm display weaker positive associations with the model predictions.

For Cooperative-Grey-PEBs (positive intention–behavior gap), Fig. 8(b1) shows that the four most important variables are, in order, attitude (E), ascription of responsibility (B), age, and knowledge (A). Figures 8(b2) and 8(b3) indicate that attitude is positively associated with higher predicted probabilities of Cooperative-Grey-PEBs when values exceed 4, with the association reversing at approximately 4.5 in the model predictions. Ascription of responsibility exhibits a similar but weaker pattern. Age shows a positive association with predicted Cooperative-Grey-PEBs up to a value of 2, after which the association remains relatively stable. Knowledge displays a negative association with predicted Cooperative-Grey-PEBs at moderate levels (3–4) and a positive association when values exceed 4.

As shown in Fig. 8(c1), the four most important variables associated with Negative-Grey-PEBs (negative intention–behavior gap) are, in order, attitude (E), ascription of responsibility (B), self-efficacy (D), and infrastructure (G). Figures 8(c2) and 8(c3) indicate a threshold-like pattern for attitude in the model predictions, with positive associations observed up to approximately 4.5 and negative associations beyond this value. Ascription of responsibility shows a similar nonlinear association, with the direction of the association changing around a value of 3.5. Higher levels of self-efficacy (4–5) are associated with lower predicted probabilities of Negative-Grey-PEBs. Infrastructure displays a positive association with predicted Negative-Grey-PEBs when values exceed 3.

Figure 8(d1) shows that the four variables most strongly associated with Red-PEBs are, in order, attitude (E), ascription of responsibility (B), self-efficacy (D), and subjective norm (C). Model predictions in Figs. 8(d2) and 8(d3) indicate that higher values of these variables (above 4) are associated with lower predicted probabilities of Red-PEBs, with attitude displaying the strongest negative association.

## Discussion

Empirical analysis supports the feasibility of the color-coded pro-environmental behavior model for identifying patterns associated with different intention–behavior configurations. For clarity, this study refers to the Cooperative-Grey group (high PEBs and low intention) and the Negative-Grey group (low PEBs and high intention) as the positive and negative intention–behavior gaps, respectively.

### Discussion of influential factors in the four groups

For the Green-PEBs group, attitude emerged as the most important variable in the model predictions, with a substantially higher contribution than self-efficacy, ascription of responsibility, and subjective norm. This pattern is broadly consistent with the Theory of Planned Behavior^34^, while also indicating a nonlinear association between attitude and the predicted probability of Green-PEBs. Specifically, the model suggests a threshold-like pattern, with stronger positive associations observed when attitude values exceed approximately 4. The comparatively weaker associations observed for self-efficacy and ascription of responsibility may be related to limited within-group variability, as these factors appear to show more pronounced positive associations with predicted Green-PEBs only at higher levels (> 4). Subjective norm exhibits a weaker association with predicted Green-PEBs, which is consistent with prior descriptions of environmentally engaged individuals as relying more on intrinsic motivations than on perceived social pressure^71^.

For the Cooperative-Grey-PEBs group, attitude emerges as the most important variable in the model predictions, displaying an inverted U-shaped association pattern. Specifically, when the attitude score exceeds the 4.5 threshold, the predictive effect reverses sharply from positive to strongly negative. This paradoxical phenomenon may be closely related to the cognitive dissonance theory^72^, in which individuals with high attitudes lack corresponding pro-environmental intentions, and their unconscious pro-environmental behaviors may trigger self-conflict, which in turn triggers psychological compensatory mechanisms, such as the conscious reduction of subsequent pro-environmental behavior. A similar but weaker inverted U-shaped association is observed for ascription of responsibility, which may be interpreted as indicating that responsibility is perceived more as an external obligation than as an internalized value within this group. Age shows a stable positive association with predicted Cooperative-Grey-PEBs up to a value of 2 (corresponding to the 25–34 age range), after which the association levels off, a pattern that may be consistent with role-based explanations emphasizing social and life-cycle expectations rather than intrinsic motivation^73^. Knowledge displays a bidirectional association pattern: moderate levels (3–4) are associated with lower predicted probabilities, whereas higher levels (> 4) are associated with higher predicted probabilities. This pattern is consistent with theoretical notions of cognitive overload and knowledge empowerment thresholds^74^.

For the Negative-Grey-PEBs group, attitude remains the most important variable in the model predictions and exhibits an inverted U-shaped association pattern. The predicted probability of Negative-Grey-PEBs increases with attitude up to approximately 4.5, after which the association reverses in the model outputs. This pattern may reflect an exploratory interpretation in which lower attitude levels are associated with weaker behavioral engagement, whereas higher attitude levels coincide with increased awareness of intention–behavior inconsistency.It is worth noting that this reversal threshold (4.5) is higher than the activation threshold (4) in the Green-PEBs population, suggesting that this group may be caught in the “high commitment trap”, where the strength of their attitudes exceeds that of the majority of environmentalists, but over-idealization of self-requirements leads to a lack of pro-environmental behaviors. Ascription of responsibility shows a nonlinear association pattern, with higher values (> 3.5) associated with increased predicted probabilities of Negative-Grey-PEBs, a pattern that may be consistent with shifts in perceived personal responsibility reported in previous studies^75^. This pattern differs from that observed for Cooperative-Grey-PEBs, highlighting heterogeneity across intention–behavior gap configurations. When self-efficacy reaches a high score band (4–5), self-efficacy shows a negative predictive effect in this group, suggesting that only under conditions of strong perceived capability does self-efficacy coincide with reduced predicted gap. Infrastructure is positively associated with predicted Negative-Grey-PEBs when values exceed 3, a pattern that is consistent with interpretations related to responsibility dilution.

For the Red-PEBs group, attitude, ascription of responsibility, self-efficacy, and subjective norm are all negatively associated with the predicted probability of Red-PEBs. This pattern contrasts with that observed for the Green-PEBs group and is broadly consistent with the Theory of Planned Behavior^34^, tin the sense that a similar set of psychological variables is associated with different intention–behavior configurations in opposite directions within the predictive framework. Notably, the consistency in the ranking of variable importance (attitude > ascription of responsibility > self-efficacy > subjective norm) across these groups highlights the central role of attitude within the model predictions. In addition, threshold-like patterns are observed for all four variables, with stronger negative associations with predicted Red-PEBs primarily emerging when variable values exceed approximately 4, which suggest nonlinear associations captured by the model.

### Limitations and future research

Although this study provides important insights, it has four main limitations: (1) Given that the sample size is insufficient to represent the entire population of Chinese residents, the findings of this study should be interpreted within the scope of the sampled population. (2) Given the imbalanced class distribution, interpretations related to specific intention–behavior gap categories should be understood as exploratory and subject to predictive limitations. (3) potential bias in questionnaire responses due to social desirability, where participants may overstate their pro-environmental intentions or behaviors. (4)The cross-sectional design prevents inference about temporal ordering or causal relationships between intention and behavior.

Future research should employ experimental or longitudinal designs and incorporate objective behavioral data to validate the observed nonlinear patterns and reduce potential measurement bias.

## Conclusions and policy implications

### Conclusions

This study explores the dual nature of the pro-environmental intention–behavior gap using an extended Theory of Planned Behavior framework and a machine learning–based analytical approach. By examining color-coded categories of pro-environmental behaviors (PEBs), several predictive and exploratory conclusions can be drawn.

Firstly, for the Green-PEBs and Red-PEBs groups, the importance rankings of attitude, ascription of responsibility, self-efficacy, and subjective norm are highly consistent across the two groups, while the directions of their associations with model predictions differ. Threshold-like patterns are observed for all four variables, with associations changing primarily when values exceed approximately 4. These findings suggest that these four psychological factors are positively associated with pro-environmental intentions and behaviors, a pattern that is broadly consistent with the Theory of Planned Behavior within a predictive framework.

Secondly, for the Cooperative-Grey-PEBs group, attitude and ascription of responsibility display inverted U-shaped association patterns in the model outputs, with predicted probabilities decreasing when values exceed approximately 4.5. And age shows a stable positive association that levels off at higher values, which may be consistent with role-based explanations (e.g., parenthood or workplace leadership). Environmental knowledge exhibits a nonlinear association pattern, with higher levels associated with increased predicted probabilities of behavior in low-intention contexts.

Thirdly, for the Negative-Grey-PEBs group, attitude shows a threshold-like association with predicted group membership, with a reversal occurring around a value of 4.5. Ascription of responsibility and self-efficacy are associated with lower predicted probabilities of Negative-Grey-PEBs at higher levels, while infrastructure is positively associated with predicted group membership when values exceed approximately 3. These findings highlight heterogeneity across intention–behavior gap configurations and suggest that contextual and psychological factors may relate differently to distinct predictive patterns.

### Policy implications

The findings of this study suggest that influencing factors differ across different groups, indicating that a one-size-fits-all policy approach may be insufficient. In light of the findings presented in Sect. 4 and Sect. 5, this paper offers policy implications tailored to distinct groups (refer to Fig. 5). Details are as follows.

At a general level, attitude emerges as a central factor in the model predictions across groups, suggesting that public awareness campaigns and values-oriented environmental education may be relevant areas for policy consideration. Such initiatives could hypothetically support stronger pro-environmental engagement.

For the Red-PEBs group, whose predicted behaviors are associated with higher perceived difficulty, policy measures that reduce practical barriers—such as providing hands-on guidance for waste sorting or simplifying pro-environmental actions—may be considered as potential supportive strategies.

For the Cooperative-Grey group, whose predicted behaviors appear to be shaped by contextual and role-related factors, policy approaches that leverage social roles, such as integrating environmental considerations into childcare programs or workplace leadership frameworks, could be explored as illustrative options. In addition, strengthening environmental knowledge dissemination may help sustain pro-environmental behaviors in low-intention contexts.

For the Negative-Grey group, predictive associations point to the potential relevance of responsibility attribution and contextual design. Hypothetical measures that clarify individual responsibilities within shared infrastructure settings—such as enhanced responsibility signaling or feedback mechanisms—may warrant consideration, while recognizing that such approaches require careful ethical and causal evaluation.

For the Green-PEBs group, policy efforts may focus on consolidating existing behaviors and encouraging peer diffusion, for example by supporting voluntary initiatives or ambassador-style programs that promote sustainable practices.

## Supplementary Information

Below is the link to the electronic supplementary material.

Supplementary Material 1

## Data Availability

All data included in this study are available upon request by contacting the corresponding author.
