# Supplementary material for: Harnessing machine learning to explore influencing mechanism in the dual pro-environmental intention-behavior gap
Source: Sci Rep. 2026 Mar 5;16:12082. doi: 10.1038/s41598-026-42468-1 (PMC13077056; doi:10.1038/s41598-026-42468-1)
Supplement: Supplementary file 1 — Supplementary Material 1 [file 41598_2026_42468_MOESM1_ESM.docx]

Supplementary Material

# S1 Supplementary Table

**Table S1.1 The scales and related literature**

| Serial number | Variables | Items | reference |
| --- | --- | --- | --- |
|  | Dependent variables | | |
|  | Behavior | (1) I always turn off the lights, and turn off the computer or TV when I'm done. |  |
|  |  | (2) I "order as much as you can eat" to minimize food waste. |  |
|  |  | (3) I always recycle used newspapers, recycle water, and print on both sides of the paper. |  |
|  |  | (4) I always promote environmental protection in my home, workplace and community and encourage others to take environmental action. |  |
|  | Intention | (1) I am willing to implement pro-environmental behaviors in my life. |  |
|  |  | (2) I intend to implement pro-environmental behaviors in my life. |  |
|  | Independent variables | | |
| A | Knowledge | (1) I understand carbon neutrality, peak carbon, and related low-carbon policies. | (Liu et al., 2020) |
|  |  | (2) I understand what exactly pro-environmental behavior entails. |  |
|  |  | (3) Turning off lights, computers, and televisions is an environmentally friendly behavior. |  |
|  |  | (4) Reducing food waste is an environmentally friendly behavior. |  |
| B | Ascription of responsibility | (1) I think my pro-environmental behavior is useful and meaningful to individuals, the state, and the community. |  |
|  |  | (2) The use of motorized vehicles for travel has a greater impact on the environment than other modes of travel, and it is the moral and duty of every citizen to reduce the use of motorized vehicles. |  |
|  |  | (3) I feel it is my responsibility to promote pro-environmental behaviors to those around me. |  |
| C | Subjective norm | (1) I think there is more social pressure to commit pro-environmental behaviors. |  |
|  |  | (2) I take advice from friends and family when practicing pro-environmental behaviors. |  |
| D | Self-efficacy | (1) I consider myself capable of implementing pro-environmental behaviors. | (Nurul Alam et al., 2023) |
|  |  | (2) Pro-environmental behavior is not a matter for one person, it needs to be practiced by everyone together. |  |
| E | Attitude | (1) I think it's necessary to implement pro-environmental behaviors. | (Ertz et al., 2016) |
|  |  | (2) I believe I have an obligation to implement pro-environmental behaviors. |  |
| F | Emotion | (1) I don't want others to engage in high-carbon behaviors. | (Zelenski and Desrochers, 2021) |
|  |  | (2) I'm more passionate about low-carbon lifestyles and pro-environmental behaviors. |  |
| G | Infrastructure | (1) The improvement of low-carbon supporting infrastructure will facilitate my implementation of pro-environmental behaviors. |  |
|  |  | (2) More comfortable, convenient, and fast public transportation would make me more inclined to pro-environmental trips. |  |
|  |  | (3) Pro-environmental infrastructure such as garbage collection and disposal is well-developed in my area. |  |
| H1 | Policies | (1) Governmental guidance policies or measures encourage my involvement in pro-environmental behaviors. |  |
| H2 |  | (2) Mandatory environmental policies or measures encourage my involvement in pro-environmental behaviors. |  |
| H3 |  | (3) Government incentives and subsidy policies or measures encourage my involvement in pro-environmental behaviors. |  |
| H4 |  | (4) Punitive environmental policies or measures encourage my involvement in pro-environmental behaviors. |  |

**Table S1.2 Demographics of respondents**

| Variables | | Frequency | Percentage (%) |
| --- | --- | --- | --- |
| Gender | Male | 965 | 43.5 |
|  | Female | 1251 | 56.5 |
| Age | < 25 | 824 | 37.2 |
|  | 25 — 34 | 735 | 33.2 |
|  | 35 — 44 | 324 | 14.6 |
|  | 45 — 55 | 224 | 10.1 |
|  | > 55 | 109 | 4.9 |
| Monthly personal income (RMB) | < 2000 | 726 | 32.8 |
|  | 2000 — 3000 | 329 | 14.8 |
|  | 3000 — 6000 | 518 | 23.4 |
|  | 6000 — 10000 | 392 | 17.7 |
|  | > 10000 | 251 | 11.3 |
| Educational level | Primary education or less | 226 | 10.2 |
|  | Junior high school | 429 | 19.4 |
|  | High school or technical secondary school | 518 | 23.4 |
|  | College and undergraduate | 592 | 26.7 |
|  | Postgraduate | 451 | 20.3 |
|  | Total | 2216 | 100 |

| **Table S1.3 Feature selection results** | | | | |
| --- | --- | --- | --- | --- |
| Features | Decision | Features | Decision | |
| A | confirmed | H2 | confirmed | |
| B | confirmed | H3 | confirmed | |
| C | confirmed | H4 | confirmed | |
| D | confirmed | Age | confirmed | |
| E | confirmed | Income | confirmed | |
| F | confirmed | Education | confirmed | |
| G | confirmed | Gender | rejected | |
| H1 | confirmed |  |  | |

# S2 Methods of Machine Learning

**Tree Structure Based Model.** First, Decision Tree is a model that performs classification or regression based on a tree structure. It recursively splits the data by selecting the best feature that reduces the impurity of the dataset (using metrics like Gini Index or Information Gain). For detailed calculations, please refer to equations (4) and (5).

Gini Index：

$Gini\left( D \right)=1-\sum_{i=1}^{C} p_{i}^{2}$(4)

where $p_{i}$ is the proportion of class $i$ in dataset $D$, and$C$is the number of classes.

Information Gain:

$IG(D,A)=Entropy(D)-\sum_{v\in A} \frac{|D_{v}|}{|D|}Entropy(D_{v})$ (5)

where $Entropy\left( D \right)=-\sum_{i=1}^{C} p_{i}\log_{2} \left( p_{i} \right),$ and $D_{v}$​ is the subset of $D$ after splitting based on feature $A$ with value $v$.

Second, LightGBM is an efficient implementation of Gradient Boosting Decision Trees (GBDT). It uses an additive model to improve prediction results through iterative optimization. LightGBM introduces histogram-based algorithms to improve computational efficiency, especially for large datasets. Refer to equations (6)-(8) for details.

Third, XGBoost is an optimized version of gradient boosting that introduces regularization terms to reduce overfitting and uses an incremental approach to iteratively refine the model. XGBoost is known for its computational efficiency and high performance in machine learning competitions. Refer to equations (6) and (7) for details.

Objective Function:

$\mathcal{\mathcal{L}}\left( \theta\right)=\sum_{i=1}^{N} \mathcal{l}\left( y_{i},\hat{y}_{i} \right)+\sum_{k=1}^{K} \Omega\left( f_{k} \right)$ (6)

where $\mathcal{l}\left( y_{i},\hat{y}_{i} \right)$is the loss function, and $\Omega\left( f_{k} \right)$ is the regularization term for the $K$-th tree.

Gradient Boosting:

$g_{i}^{\left( t \right)}=\frac{\partial\mathcal{l}\left( y_{i},\hat{y}_{i}^{\left( t-1 \right)} \right)}{\partial\hat{y}_{i}}$ (7)

$h_{i}^{(t)}=\frac{\partial^{2}\mathcal{l(}y_{i},\hat{y}_{i}^{(t-1)})}{\partial\hat{y}_{i}^{2}}$(8)

where $g_{i}^{\left( t \right)}$​ and $h_{i}^{(t)}$ are the first and second derivatives of the loss function in iteration $t$.

Fourth, Random Forest is an integrated learning method that trains multiple decision trees and obtains the final prediction by voting or averaging. Each tree is trained on randomly selected samples and features to avoid overfitting.

**K-Nearest Neighbors (KNN).** KNN is a distance-based classification method that determines the class of a sample by finding the $k$ nearest neighbors using a distance metric (such as Euclidean distance) and assigning the most frequent class among those neighbors. Refer to equation (9) for details.

Euclidean Distance:

$d\left( x,y \right)=\sqrt{\sum_{i=1}^{n} (x_{i}-y_{i})^{2}}$(9)

where $x$ and $y$ are two samples, and $n$ is the number of features.

The class prediction is determined by the majority class of the $k$ nearest neighbors.

**Multilayer Perceptron (MLP).** The Multilayer Perceptron (MLP) in this study is a simple neural network with a single hidden layer. The network consists of an input layer, one hidden layer, and an output layer. Each layer is fully connected to the next, and the output of each layer is determined by a weighted sum of the inputs, followed by the application of an activation function. Refer to equations (10)-(12) for details.

Forward Propagation:

$h^{(1)}=f(W^{(1)}X+b^{(1)})$(10)

where$h^{(1)}$is the output of the hidden layer, $W^{(1)}$ is the weight matrix of the hidden layer, $X$ is the input, and $b^{(1)}$ is the bias term. $f$ is the activation function, such as ReLU or Sigmoid.

The output of the network is:

$\hat{y}=f(W^{\left( 2 \right)}h^{\left( 1 \right)}+b^{\left( 2 \right)})$(11)

Where $\hat{y}$ is the predicted output, $W^{\left( 2 \right)}$ is the weight matrix of the output layer, and $\mathbf{b}^{\left( 2 \right)}$ is the bias term.

Loss Function (Cross-Entropy Loss):

$\mathcal{\mathcal{L}}\left( \theta\right)=-\sum_{i=1}^{N} y_{i}\log\left( \hat{y}_{i} \right)+\left( 1-y_{i} \right)\log\left( 1-\hat{y}_{i} \right)$(12)

where $y_{i}$ is the true label, and $\hat{y}_{i}$​ is the predicted value.

This architecture with a single hidden layer allows the network to capture non-linear relationships between the input features and the output.

**Lasso Regression.** Lasso (Least Absolute Shrinkage and Selection Operator) is a linear regression method that introduces an L1 regularization term to the loss function. This regularization term penalizes the absolute values of the regression coefficients, encouraging sparsity in the model by shrinking some coefficients to zero. It is particularly useful for feature selection in high-dimensional datasets. Refer to equation (13) for details.

Objective Function:

$L(\theta)=\sum_{i=1}^{N} (y_{i}-\hat{y}_{i})^{2}+\lambda\sum_{j=1}^{p} |\beta_{j}|$ (13)

where $y_{i}$is the true value, $\hat{y}_{i}$ is the predicted value, $\beta_{j}$​ is the regression coefficient for feature $j$, $p$ is the number of features, and $\lambda$ is the regularization parameter controlling the strength of the penalty.

**Multinomial Logistic Regression (MLR).** Multinomial Logistic Regression is a generalization of binary logistic regression used for multi-class classification problems. It estimates the probability that a sample belongs to each class using the SoftMax function. MLR assumes independence among the classes and models the log-odds of each class relative to a reference class. Refer to equations (14)–(16) for details.

SoftMax Function:

$P(y_{i}=k|x_{i})=\frac{e^{\beta_{k}^{T}x_{i}}}{\sum_{j=1}^{K} e^{\beta_{j}^{T}x_{i}}}$ (14)

where $x_{i}$ is the feature vector of the $i$-th sample, $\beta_{k}$​ is the coefficient vector for class $k$, and $K$ is the total number of classes.

Prediction Rule:

$\hat{y}_{i}=\arg\max_{k} P\left( y_{i}=k | x_{i} \right)$ (15)

Loss Function (Cross-Entropy Loss):

$L(\theta)=-\sum_{i=1}^{N} \sum_{k=1}^{K} y_{ik}\log P(y_{i}=k|x_{i})$ (16)

Where $y_{ik}=1$ if sample $i$ belongs to class $k$, otherwise $0$.

**Model Evaluation.** The model evaluation outputs all the classification prediction model evaluation metrics, of which the more commonly used ones include accuracy, precision, recall, F1 score and AUC value. The specific calculation methods are shown in Equations (17) through (20). For each model, the performance on the test set will be evaluated by these metrics. In addition, the generalization ability of the model is further evaluated using the k-Fold Cross Validation method, which ensures the robustness of the model under different data divisions. In k-Fold Cross Validation, the dataset is divided into k subsets, with k-1 subsets being used for training each time and the remaining one subset being used for validation. By repeatedly training and validating the model, the performance of the model on different data subsets can be obtained, thereby enabling a more accurate assessment of the model's generalization ability.

$Accuracy=\frac{\text{Number of Correctly Classified Samples}}{\text{Total Number of Samples}}$(17)

$Precision=\frac{\text{True Positives}}{\text{True Positives}+\text{False Positives}}$(18)

$Recall=\frac{\text{True Positives}}{\text{True Positives}+\text{False Negatives}}$(19)

$F1=2\times\frac{Precision\times Recall}{Precision+Recall}$(20)
